# Abuse of Medical Charities, and Unprofessional Conduct of Clinical Examiners

**Published:** 1880-03

**Authors:** Alfred L. Carrol

**Affiliations:** New Brighton, N. Y.


### ABUSE OF MEDICAL CHARITIES, AND UNPROFESSIONAL CONDUCT OF CLINICAL EXAMINERS.

*To the Editor of The Medical Record.*—Sir:—The letter of Dr. Seabury Jones, in your issue of February 14, touches an abuse of which glaring examples are almost daily occurring; and if the time be ripe for its discussion, I beg leave to contribute to the harmony of the occasion a few out of many illustrative instances which have fallen under my notice. Names, dates, and places are suppressed for the same reasons that Dr. Jones gives.

1. A boy, the son of well-to-do trades-people, suffered a comminuted fracture of the lower end of the humerus, extending into the elbow joint, and giving rise to severe synovitis. He was attended by a thoroughly competent physician, who, in view of the gravity of the case, called in consultation Dr. W. C. Anderson, a surgeon whose reputation is familiar to most of your older readers, and, under the joint care of these gentlemen, recovery ensued with a more useful joint than is commonly seen after similar injuries. Some months afterward, the parents, incited by village gossips, took the patient to a college clinic in New York, whereat the officiating "Professor" is alleged to have stated that the case had been ill-treated, and that the only resource to escape crippling for life was to let him, the said "Professor," refracture the arm and set it properly. Making every allowance for possible exaggeration on the part of the parents, it is certain that this adverse opinion was forcibly enough expressed to induce them to threaten a suit for malpractice, and consequently I was asked to examine the boy. There had apparently been a transverse fracture, with separation of the condyles of the humerus; a good deal of exudation and induration existed about the joint; but the general relations of the parts were well preserved, and he could easily place his hand upon the back of his head, and extend his arm to an angle of about 130°; a result which I think most surgeons would deem very satisfactory, considering the character of the injury, and the history of the case. Not only was grave injustice done to an educated and painstaking physician in this case, but the people to whom such a damaging opinion was given, were in no respect fit recipients of "advice gratis."

2. A patient under the care of one of my friends, who is a skilled gynecologist, was induced to apply at a well-known special hospital, her trouble being an uncomplicated anteversion of the uterus. One of the assistant surgeons made an examination, introduced a pessary, and cautioned her on no account to permit any "country doctor" to treat her, as none of them knew how to use such instruments.

3. An elderly woman, with a forward luxation of the shoulder, was attended by a neighboring practitioner shortly after the accident. Much inflammation and pain supervening, I saw the case in consultation, and satisfied myself that the luxation had been reduced, and adequate retentive means adopted. As is usual among the lower classes, it appears that her physician's directions were neglected, and she passed from under his observation for some weeks, when she again "turned up," with a menace of a suit for damages, based upon the alleged opinion of another assistant surgeon at a city hospital, who, as she averred, told her that her shoulder had never been properly "put in joint." I may add, that about two months after her hospital treatment, she was admitted to the Smith Infirmary, on Staten Island, where I assisted in again reducing the luxation, the head of the humerus being firmly fixed under the clavicle. I have little doubt that since then the displacement may have recurred, and afforded further occasion for a hospital criticism on surburban surgery.

Aside from such explicit or implied condemnation of private practitioners (which is not wholly confined to hospital and college precincts), it occasionally happens that "out-departments" and clinics are made to conduce to the pecuniary profit of some of the officiating staff, where inquiry has shown that the patient is not in destitute circumstances, but has not been pushed to the inquisitorial extent of ascertaining whether the case were already under treatment or not. More than a few office fees have been drafted from college waiting-rooms by shrewd young gentlemen who were fortunate enough to be on the "clinical staff" of the attending professor; indeed I was once informed by one of these rising lights that the facilities for thus building up a paying practice constituted the chief attraction of his position. I have even known of a distinguished professor himself transferring from his clinic to his private emolument a patient whom he found able to pay a fee, and who had been paying fees to another physician. And dispensaries offer a still wider field for this sort of exploration: *e.g.*, a mechanic's wife, having an elongated cervix uteri and "pin-hole" os, went to one of the up-town dispensaries for advice. The physician told her that her case required more attention than he could give to it at the dispensary, and required her to come to his office, not omitting to mention his charge per visit. She went to him several times (paying a fee on each occasion), and was treated by means of a tonic mixture and the introduction of a Smith's pessary, for which he charged her one dollar extra (a profit of 100 per cent.). It is scarcely necessary to observe that, beyond an increase of vaginal leucorrhœa, no great alteration of the morbid condition was effected by this means.

Examples of both forms of abuse of medical charity might be multiplied; but I have trespassed far enough upon your space, and leave to others the extension of the list.

I am, sir, yours, etc.,

ALFRED L. CARROL.

NEW BRIGHTON, N. Y., February 20, 1880.

